# Probio‐M9, a breast milk‐originated probiotic, alleviates mastitis and enhances antibiotic efficacy: Insights into the gut–mammary axis

**DOI:** 10.1002/imt2.224

**Published:** 2024-07-09

**Authors:** Jie Yu, Weicheng Li, Ruibo Xu, Xiaoye Liu, Guangqi Gao, Lai‐Yu Kwok, Yongfu Chen, Zhihong Sun, Wenjun Liu, Heping Zhang

**Affiliations:** ^1^ Key Laboratory of Dairy Biotechnology and Engineering, Ministry of Education Inner Mongolia Agricultural University Hohhot China; ^2^ Key Laboratory of Dairy Products Processing, Ministry of Agriculture and Rural Affairs Inner Mongolia Agricultural University Hohhot China; ^3^ Inner Mongolia Key Laboratory of Dairy Biotechnology and Engineering Inner Mongolia Agricultural University Hohhot China; ^4^ Collaborative Innovative Center for Lactic Acid Bacteria and Fermented Dairy Products, Ministry of Education Inner Mongolia Agricultural University Hohhot China; ^5^ School of Biological Science and Food Engineering Chuzhou University Chuzhou China

## Abstract

Breast milk naturally contains lactic acid bacteria, but their precise origin remains a subject of debate. In this study, we utilized a rat mastitis animal model to investigate the potential of a breast milk‐derived probiotic strain, *Lacticaseibacillus rhamnosus* Probio‐M9, in alleviating mastitis and enhancing the efficacy of antibiotic treatment. Through histopathological analysis of mammary tissue, we observed that Probio‐M9 effectively relieved mastitis, mitigated inflammation, and improved the response to antibiotic treatment. Metagenomic analysis further revealed that Probio‐M9 enhanced interactions among gut microbes, accompanied by an increase in the relative abundance of *Ruminococcaceae* and the regulation of specific genes and carbohydrate‐active enzymes, subsequently impacting host immunity. Additionally, an intriguing finding was the translocation of live Probio‐M9 from the gut to the mammary tissue only during bacterial mastitis and lactation, likely facilitated through lymphatic circulation. These findings advance our understanding of the intricate gut–mammary axis and provide valuable insights into the potential health benefits of probiotic interventions.

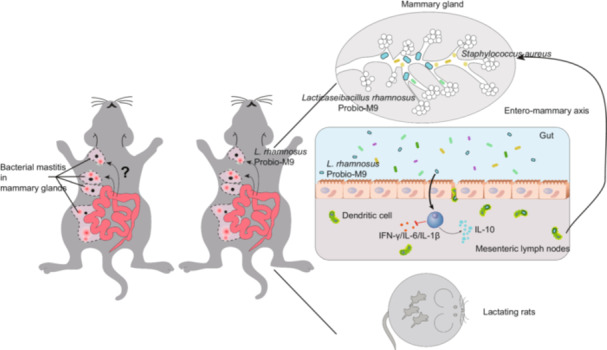

Breast milk, widely recognized as the optimal nutrition for infants, plays a vital role in promoting their health [1, 2]. While breast milk was traditionally thought to be sterile, advances in culture techniques have enabled the isolation of various microorganisms associated with breast milk [3, 4]. Understanding the role of breast milk microbiota in maternal–infant health has become an emerging research focus, with breast milk being acknowledged as a potential source of probiotics [5, 6]. However, the origin of these microorganisms in breast milk remains an intriguing question that requires further investigation [3].

Mastitis is an inflammatory reaction of the mammary gland, and it is a prevalent condition that affects nearly all lactating mammals [7]. Mastitis leads to substantial economic losses worldwide due to the significant reduction in milk quality and quantity, directly impacting profitability and productivity [8, 9]. It has been suggested that breast milk probiotics can be transmitted to newborns through breastfeeding [10]. In pregnant mice, orally administered *Lactococcus lactis* MG1614 and *Ligilactobacillus salivarius* PS2 have been observed to subsequently be detected in their mammary tissue and milk [11]. A similar phenomenon was observed in lactating rats supplemented with *Limosilactobacillus fermentum* CECT5716 for 5 weeks [12]. However, it remains unclear whether the translocation of gastrointestinal bacteria to the mammary gland is a specific or random process [13].

In this study, we evaluated the efficacy of the probiotic strain, *Lacticaseibacillus rhamnosus* Probio‐M9, in treating mastitis by using a rat mammary model infected with *Staphylococcus aureus*. Our findings demonstrate that Probio‐M9 has both preventive and therapeutic effects on mastitis. Furthermore, our study suggests the existence of a pathway of the gut–mammary translocation of Probio‐M9.

## RESULTS

### Experiment I, Probio‐M9 augmented the prophylactic and therapeutic effects of cephalexin treatment for bacterial mastitis

**Figure 1:**
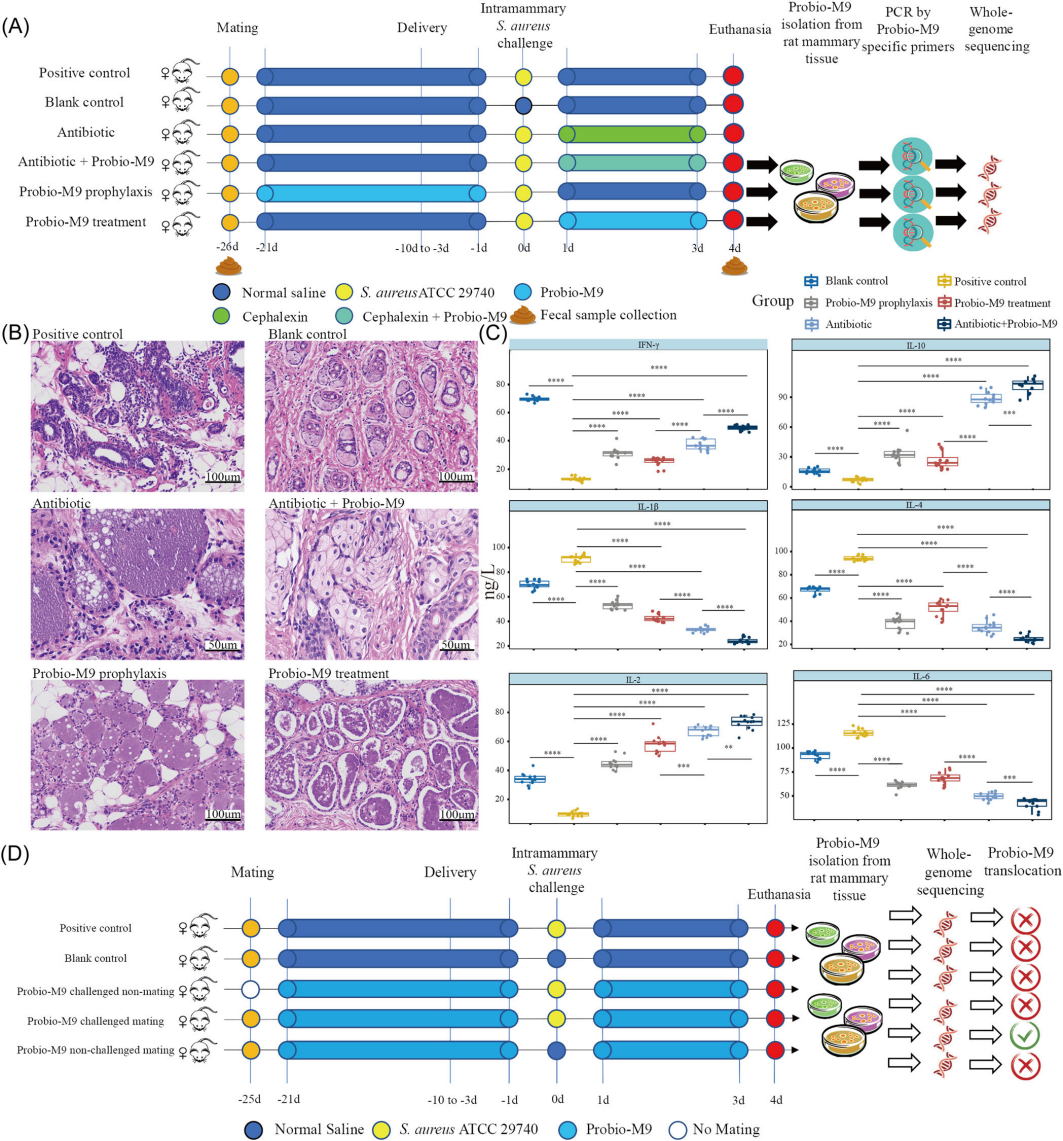
Experimental design of Experiments I and II and impact of *Staphylococcus aureus* challenge and Probio‐M9 treatment on mammary tissue pathology and cytokine levels in Experiment I. (A) In this experiment, 72 female rats were randomly divided into six groups: positive control, blank control, antibiotic, antibiotic + Probio‐M9, Probio‐M9 treatment, and Probio‐M9 prophylaxis. After mating, the female rats were housed in individual cages. Except for the blank group, all lactating rats in other groups were subjected to intramammary challenge with *S. aureus* ATCC27940 3 days after delivery. The antibiotic group and the antibiotic + Probio‐M9 group were treated with cephalexin, while the Probio‐M9 treatment group and antibiotic + Probio‐M9 group were administered Probio‐M9 through feeding. The Probio‐M9 prophylaxis group received Probio‐M9 for 20 days after copulation. On the fourth day after the challenge, all female rats were euthanized. The mammary glands of rats that were fed Probio‐M9 were collected, homogenized, cultured, and subjected to DNA extraction. Probio‐M9‐specific primers were used to confirm strain identity, followed by whole‐genome sequencing and comparative genomic analysis. (B) Representative mammary gland tissue sections of euthanized rats in different groups, stained by hematoxylin and eosin (×200 or ×400 magnification). (C) Boxplots showing the intramammary levels of six cytokines, interferon (IFN)‐γ, interleukin (IL)‐4, IL‐10, IL‐1β, IL‐2, and IL‐6. Significant differences were detected using the Wilcoxon test. **p < 0.01, ***p < 0.001, ****p < 0.0001**.** (D) Experiment II was conducted to investigate the translocation of Probio‐M9 homologous isolates from the gut to mammary tissue following lactation and *S*. *aureus* challenge. Sixty rats were randomly divided into five groups: positive control, blank control, Probio‐M9 challenged nonmating, Probio‐M9 challenged mating, and Probio‐M9 nonchallenged mating groups. PCR, polymerase chain reaction.

In Experiment I, we evaluated the therapeutic effect of Probio‐M9 on *S. aureus*‐induced mastitis in rats by analyzing the pathological and immune responses in the mammary gland tissues (Figure 1B,C). In contrast, the positive control group, which was challenged with *S. aureus*, exhibited complete detachment of the mammary epithelium, disrupted tissue structure, dense infiltration of inflammatory cells, and suppurative lesions. The Probio‐M9 prophylactic group showed lymphocyte infiltration in the acinar cavity and stroma. The Probio‐M9 treatment group exhibited increased lymphocyte infiltration in the mammary gland, accompanied by reduced acinar secretory ability and size of mammary gland alveoli. The antibiotic treatment group displayed significant lymphocyte infiltration in the acinar cavity, with disorganized acinar cells and an increased number of migrating immune cells in the acinar wall. The Probio‐M9 and antibiotics combined treatment group exhibited pronounced lymphocyte infiltration in the acinar cavity and a higher number of immune cells translocating from the acinar wall. Then, we analyzed the cytokine levels in the mammary tissues of rats. On Day 4 of the bacterial challenge, mastitis animals in the positive control group exhibited significantly elevated levels of interleukin (IL)‐4, IL‐1β, and IL‐6 (*p* < 0.05 in all cases; Figure 1C) and significantly decreased levels of interferon (IFN)‐γ, IL‐2, and IL‐10 (*p* < 0.05) compared to the blank control group. This was evidenced by the significant reduction in the levels of pro‐inflammatory cytokines, IL‐1β and IL‐6, in the mammary gland tissue of the rats (*p* < 0.05) and the significant increase in the anti‐inflammatory cytokine IL‐10 level (*p* < 0.05), compared to the positive control group. The administration of cephalexin showed significantly better results compared to probiotics alone, whether used prophylactically or as a treatment (*p* < 0.05).

**Figure 2:**
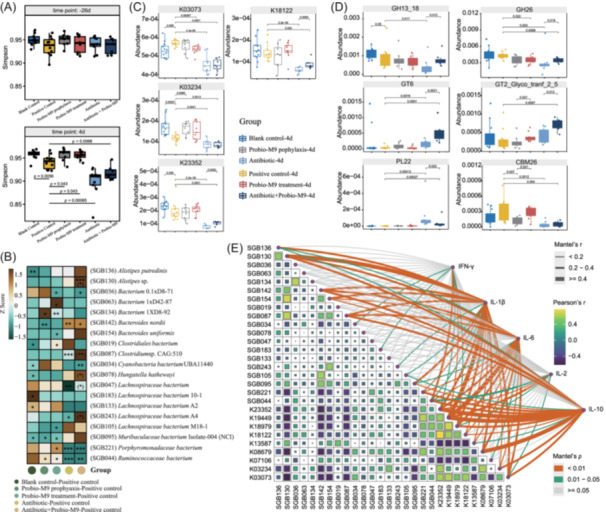
Comparative analysis of fecal microbiota and fecal functional metagenome of different rat groups in Experiment I. (A) Boxplots showing the Simpson diversity index of the fecal microbiome at baseline (−26 days, baseline) and 4 days after *Staphylococcus aureus* challenge (4 days) in six groups, respectively. Statistical differences in the Simpson diversity index between groups were evaluated with unpaired Wilcoxon tests, and significant *p* values were indicated. (B) Heatmap showing the relative abundance of differentially abundant species‐level genome bins (SGBs) between groups at 4 days. Baseline SGB abundance showed no significant differences between groups, but significant changes occurred only after the intervention. The color scale represents the *Z* score in the comparative abundance analysis, ranging from high abundance (brown) to low abundance (blue‐green). Significant differences between the positive control group and other groups are represented by *p < 0.05, **p < 0.01, and ***p < 0.001; between the antibiotic group and the antibiotic + Probio‐M9 are represented by *p < 0.05 and **p < 0.01 in parentheses. (C, D) Boxplots showing significant differential Kyoto Encyclopedia of Genes and Genomes Orthologies (KOs) and carbohydrate‐active enzymes, including glycoside hydrolases (GH), glycosyltransferases (GT), polysaccharide lyases (PL), and their appended noncatalytic carbohydrate‐binding modules between groups. All the shown KOs and carbohydrate‐active enzymes were not significantly different between groups at baseline. The indicated *p* values represent statistically significant differences (cut‐off level of *p* < 0.05, Wilcoxon test). (E) The heatmap illustrates Pearson's correlation between significant differential abundant KOs and SGBs, while the network shows the correlations between cytokine levels, including interferon (IFN)‐γ, interleukin (IL)‐10, IL‐1β, IL‐2, and IL‐6, and these KOs and SGBs, evaluated by the partial Mantel test. Mantel's *p* and *r* values are indicated by different line colors and thickness, respectively, while Pearson's correlation is represented by the color scale.

In Experiment I, the fecal metagenomes of rats were analyzed 26 days before mammary gland injection (−26 days) and 4 days after mammary gland injection (4 days; Figure 1A). No significant intergroup differences were observed in fecal microbiota diversity (measured by the Simpson's diversity index) among all groups at −26 days (*p* > 0.05; Figure 2A). At 4 days, the positive control group exhibited significantly lower alpha diversity compared to the blank control group. However, this reduction could be mitigated by probiotic intake (*p* < 0.05). While antibiotics effectively relieved mastitis symptoms in rats, they also reduced the host gut microbiota diversity (*p* < 0.01).

We then analyzed the species‐level genome bins (SGBs) in the fecal metagenomes at 4 days after mammary gland injection (Table S1; Figure 2B). Principal coordinate analyses demonstrated significant differences between the antibiotic groups and the positive control group (Figure S1). However, no differences were observed in the fecal microbiota structure between the positive control group, Probio‐M9 prophylaxis group, and Probio‐M9 treatment group (Figure S1). Nineteen SGBs showed significant differences between groups (*p* < 0.05; Figure 2B). The abundance of *Bacteroides nordii* and *Muribaculaceae* bacterium isolates‐004 (NCI) also decreased significantly in the probiotic prophylaxis group compared to the positive control group (*p* < 0.05). In comparison to the antibiotic group, the probiotic antibiotic treatment group exhibited a significant increase in the abundance of *Alistipes* sp., *Clostridium* sp. CAG:510, *Hungatella hathewayi*, *Lachnospiraceae* bacterium, and *Lachnospiraceae* bacterium A4 (*p* < 0.05). To better understand the interaction within the gut microbial community of the six groups of rats, we performed a co‐occurrence network analysis at 4 days (Figure S2). The gut microbial correlation networks of the probiotic treatment group and the prophylaxis group demonstrated more node count and edge count compared to the positive control group (Tables S2 and S3).

Compared to the blank control group, rats with mastitis exhibited significant alterations in the gene abundance of specific Kyoto Encyclopedia of Genes and Genomes Orthologies (Figures 2C,D and S3) and carbohydrate‐active enzymes, including K03234, and K03073. However, administration of probiotics significantly alleviated some of these changes (*p* < 0.05, Figure 2C, Table S4). Additionally, when comparing the antibiotic group to the antibiotic + Probio‐M9 group, there was a significant increase in the gene abundance of K18122 and K23352 (*p* < 0.01). Moreover, the levels of GH13_18, GH26, GT6, and GT2_Glyco_tranf_2_5 in the antibiotic + Probio‐M9 group were significantly higher than in the antibiotic group (*p* < 0.05; Figure 2D, Table S5). Conversely, the level of PL22 in the antibiotic + Probio‐M9 group was significantly lower than in the antibiotic group (*p* < 0.05).

Subsequently, we analyzed the correlation between the relative abundance of SGBs and the differential genes and inflammatory factors (Table S6 for *p* values; Table S7 for *R* values). We found that the relative abundance of *Clostridium* sp. CAG:510, which was more abundant in the antibiotic + Probio‐M9 group, showed a positive association with the K13587 (*p* < 0.05, *R* = 0.3, Figure 2E). Among the differential genes (*p* < 0.05, Figure 2E), K07106 and K13587 were positively correlated with each other (*R* = 0.4), while they exhibited negative correlations with other differential genes, such as K23352, K19449, K18979, K18122, and K03073 (*p* < 0.05, *R* < −0.3). Moreover, all five cytokines (IFN‐γ, IL‐1β, IL‐6, IL‐2, and IL‐10) showed correlations with K19449, K23352, and *Ruminococcaceae* bacterium (Figure 2E). These findings suggest that the aforementioned species and metabolic pathways may play a key role in modulating immunity. In summary, our results strongly indicate that probiotics, especially when combined with antibiotics, are effective in restoring gut microbial balance and thereby alleviating mastitis.

### Live Probio‐M9 translocated endogenously from the gut to mammary tissue

A previous study demonstrated that probiotic lactic acid bacteria ingested by pregnant women could be detected in their breast milk [11]. Therefore, we attempted to recover Probio‐M9 from the mammary tissue of rats that were fed with Probio‐M9. Thirty‐six single colonies of Probio‐M9 homologous bacteria were successfully recovered, and their identity was confirmed with strain‐specific polymerase chain reaction (PCR), whole‐genome sequencing, and comparative genomics (Table S8).

Genomic comparisons between the original Probio‐M9 and the assembled genomes of the 36 isolates from rat mammary glands revealed consistent genome sizes, with an average nucleotide identity (ANI) of 99.98% and a total nucleotide identity of 98.53% (Figure S4). Among the 36 cultured isolates, only five showed a low number (<8) of single nucleic acid polymorphisms (SNPs) (Figure S5, Table S9), they were considered to be derived from Probio‐M9. At the genetic level, 94.95% (2632) of the 2772 genes of Probio‐M9 were shared by isolates from mammary gland tissues (Table S10). The successful recovery of Probio‐M9 in the mammary gland tissue of rats that ingested the probiotic suggests that these bacteria can translocate to the mammary tissue after ingestion.

### Experiment II, translocation of Probio‐M9 only occurred during lactation and bacterial mastitis, possibly via the lymphatic circulation

We conducted Experiment II, which closely resembled Experiment I but with two key modifications. First, we excluded antibiotic treatment from the experimental design. Second, we included two additional groups: a nonmated (nonlactating) group and a group of rats not challenged with *S. aureus* but administered with Probio‐M9 (Figure 1D). The primary objective of Experiment II was to determine whether lactation and mastitis resulting from *S. aureus* infection were necessary conditions for Probio‐M9 translocation to occur. We collected and homogenized both mammary glands and mesenteric lymph nodes (MLNs) from different groups of rats. Subsequently, we cultured these samples to recover Probio‐M9 for further analysis.

A total of 85 Probio‐M9 homologous isolates were obtained from the mammary glands (43 isolates) and MLN (42 isolates; Table S11). In the Probio‐M9 challenged mating group, at least one isolate was successfully recovered from each collected mammary gland and MLN of the 12 rats (Table S11). To confirm their identity, these isolates underwent strain‐specific PCR and whole‐genome sequencing. The ANI between these isolates and Probio‐M9 was found to be 99.97% (Figure S6). Importantly, all 85 isolates had a high level of relatedness between these 85 isolates and Probio‐M9 (Figure S7A, Table S12). In total, there were only 20 SNPs across all isolates (Figure S7B, Table S12). Notably, no common mutations were found between the original Probio‐M9 strain and the 85 cultures (Table S13).

It is worth noting that all 85 cultures were isolated exclusively from lactating rats that received both the probiotic and *S. aureus*, while no Probio‐M9 homologous isolates were recovered from the mammary tissue and MLN of the unmated rat group and or the group that received Probio‐M9 alone in the absence of *S. aureus*. This observation suggests that both *S. aureus*‐induced mastitis and lactation were necessary prerequisites for bacterial translocation to occur. In addition, we can confidently assert that the observed phenomenon was not a result of nipple contamination with probiotics. This conclusion is supported by the fact that two out of three groups of rats that were fed probiotics did not exhibit this gut–mammary translocation after probiotic administration. If nipple contamination had occurred, all rats receiving probiotics would likely have shown signs of contamination.

Intriguingly, no significant differences between the isolates recovered from the MLN and mammary gland tissues, providing evidence that bacterial translocation likely occurred through the lymphatic system. A previous study has proposed that bacterial translocation from the digestive tract of healthy women could serve as a source of bacteria for the mammary gland during late pregnancy and lactation [14]. Following our observations, some interesting fundamental questions arise: (1) How are these endogenous bacterial translocations conditioned? (2) How do bacteria translocate from the gut to the mammary tissue? We hypothesize that some gut microbes might be carried by dendritic cells and subsequently translocated via immune circulation from the MLN to lymph nodes near the mammary tissue. Our hypothesis is based on the findings of a previous study in 2003, which described an endogenous pathway through the gut–mammary axis [15]. No research has yet explored the specific pathways of bacterial translocation from the MLN to mammary glands in human or animal models.

## CONCLUSION

In this study, we investigated the potential of Probio‐M9 to alleviate mastitis and mitigate the adverse effects of antibiotic therapy. Through genomic‐level analysis, we have identified instances of independent Probio‐M9 translocation from the rat gut to the mammary gland, potentially involving the lymphatic system. Moreover, we have confirmed the requirement of lactation and the development of bacterial mastitis for bacterial translocation. While the precise mechanism of probiotic translocation remains unclear, our results suggest a selective rather than random process. This study will contribute to advancing our knowledge of the entero‐mammary axis and its implications in health and disease.

## AUTHOR CONTRIBUTIONS

Heping Zhang contributed to the study concept and design. Jie Yu, Ruibo Xu, Guangqi Gao, Wenjun Liu, Yongfu Chen, and Zhihong Sun conducted data collection and gave conceptual advice for the manuscript. Lai‐yu Kwok wrote and revised this manuscript. Weicheng Li, Ruibo Xu, and Xiaoye Liu analyzed the data and prepared the figures. Jie Yu and Weicheng Li conceived, wrote, and revised this manuscript. All authors have read the final manuscript and approved it for publication.

## CONFLICT OF INTEREST STATEMENT

The authors declare no competing interests.

## ETHICS STATEMENT

The ethics application (No. [2020]042) was approved by the Research Ethics Committee of the Inner Mongolia Agricultural University.

## Supporting information


**Figure S1.** Analysis of beta diversity of fecal metagenome in rats.
**Figure S2.** Co‐occurrence networks of each group at day 4 of fecal metagenome in rat.
**Figure S3.** Analysis of metagenomic functional gene differences of fecal metagenome in rat.
**Figure S4.** Analysis of nucleic acid diversity of Probio‐M9 isolates in rats in experiment I.
**Figure S5.** Phylogenetic analysis of Probio‐M9 isolates in rats in experiment I.
**Figure S6.** Analysis of nucleic acid diversity of Probio‐M9 isolates in rats in experiment II.
**Figure S7.** Phylogenetic analysis and single nucleic acid polymorphisms (SNPs) functional annotation of rat Probio‐M9 isolates based on SNPs analysis in experiment II. 

**Table S1.** The relative abundance of bacterial species in rat gut in Experiment I.
**Table S2.** Numbers of nodes analyzed by co‐occurrence network for different groups.
**Table S3.** Numbers of edges analyzed by co‐occurrence network for different groups.
**Table S4.** The abundance of fecal microbiome genes in Experiment I.
**Table S5.** The abundance of carbohydrate‐active enzymes in Experiment I.
**Table S6.** The *p* values of comparisons of differential species‐level genome bins (SGBs) and metabolic genes in Experiment I.
**Table S7.** The *r* values of comparisons of differential species‐level genome bins (SGBs) and metabolic genes in Experiment I.
**Table S8.** Genome assembly information of isolates in Experiment I.
**Table S9.** Single nucleotide polymorphism sites of isolates in Experiment I.
**Table S10.** The presence and absence of genes in isolates in Experiment I.
**Table S11.** Genomic characterization and taxonomy of isolates in Experiment II.
**Table S12.** Single nucleotide polymorphism sites of isolates in Experiment II.
**Table S13.** The presence and absence of genes in isolates in Experiment II.

## Data Availability

The isolate assembly data from this study have been deposited in the NCBI SRA data repository under the BioProject ID number PRJNA686682. The metagenomics data have been uploaded to the NCBI SRA data repository under the BioProject ID number PRJNA902684. The data and scripts used are saved in GitHub https://github.com/lxye1720/probio-m9.git. Supplementary materials (methods, figures, tables, graphical abstract, slides, videos, Chinese translated version, and update materials) may be found in the online DOI or iMeta Science http://www.imeta.science/.
