# Supplementary material for: Probio‐M9, a breast milk‐originated probiotic, alleviates mastitis and enhances antibiotic efficacy: Insights into the gut–mammary axis
Source: Imeta. 2024 Jul 9;3(4):e224. doi: 10.1002/imt2.224 (PMC11316926; doi:10.1002/imt2.224)
Supplement: Supplementary file 1 — Figure S1. Analysis of beta diversity of fecal metagenome in rats. Figure S2. Co‐occurrence networks of each group at day 4 of fecal metagenome in rat. Figure S3. Analysis of metagenomic functional gene differences of fecal metagenome in rat. Figure S4. Analysis of nucleic acid diversity of Probio‐M9 isolates in rats in experiment I. Figure S5. Phylogenetic analysis of Probio‐M9 isolates in rats in experiment I. Figure S6. Analysis of nucleic acid diversity of Probio‐M9 isolates in rats in experiment II. Figure S7. Phylogenetic analysis and single nucleic acid polymorphisms (SNPs) functional annotation of rat Probio‐M9 isolates based on SNPs analysis in experiment II. [file IMT2-3-e224-s001.docx]

**Supporting information to**

**Probio-M9, a breast milk-originated probiotic, alleviates mastitis and enhances antibiotic efficacy: Insights into the gut-mammary axis**

**Running Title: Probio-M9: mastitis alleviation and gut-mammary translocation**

Jie Yu^1#^, Weicheng Li^1#^, Ruibo Xu^1#^, Xiaoye Liu^1#^, Guangqi Gao^1^, Lai-Yu Kwok^1^, Yongfu Chen^1^, Zhihong Sun^1^, Wenjun Liu^1^, Heping Zhang^1^*

^1^Key Laboratory of Dairy Biotechnology and Engineering, Ministry of Education; Key Laboratory of Dairy Products Processing, Ministry of Agriculture and Rural Affairs; Inner Mongolia Key Laboratory of Dairy Biotechnology and Engineering; Inner Mongolia Agricultural University, Hohhot 010018, China

^#^These authors contributed equally: Jie Yu, Weicheng Li, Ruibo Xu, Xiaoye Liu

* Correspondence: [hepingdd@vip.sina.com](mailto:hepingdd@vip.sina.com) (Heping Zhang)

**Methods**

**Bacterial strains**

*Staphylococcus aureus* ATCC 29740 was obtained from Professor Songhua Hu at Zhejiang University, China. The probiotic strain, Probio-M9, was prepared as a dry powder for administration to the animals via intra-gastric gavage. It was dissolved in 0.9% saline at a concentration of 4 ×10^9^ CFU/mL when used. The Probio-M9 strain was provided by the Key Laboratory of Dairy Biotechnology and Engineering, Ministry of Education, Inner Mongolia Agricultural University.

**Experimental animals**

Throughout the study, specific-pathogen-free animals were used and housed in a controlled environment that was also specific-pathogen-free. This environment was carefully maintained to prevent the presence of disease-causing microorganisms, thereby ensuring the reliability of research results. The animals were kept under a 12-h light-dark cycle, at a room temperature of 23 ± 2 °C, and a relative humidity of less than 65%.

Two separate rat experiments were conducted in this study. In Experiment I, a total of 72 female and 36 male Wistar rats, aged between 8-12 weeks, were used. These rats were housed in the animal facility of the Key Laboratory of Dairy Biotechnology and Engineering, Ministry of Education, China. For the purpose of mating, groups consisting of one male and two female rats were housed together in micro-isolator cages for mating to occur. The rats had access to tap water *ad libitum*, and bedding, water, and feed were changed daily. After mating, the female rats were individually kept in separate cages.

Experiment II involved 60 female and 30 male Wistar rats, aged between 8-12 weeks. These rats were provided with a sterile chow diet (Catalog number 1035, Beijing Huafukang Biology Technology Co., Beijing, China). The diet had a carbohydrate calorie ratio of 55% (detailed information regarding the composition and calorie ratio is available in Table S6).

**Experimental design and sampling**

In Experiment I (Fig. 1 for the study design), a total of six groups (n = 12 female rats per group) of postnatal (lactating) rats were included. The groups and their corresponding treatments were as follows: the positive control group, without Probio-M9, which underwent postnatal *S. aureus* challenge (n = 11); the blank control group, without Probio-M9 and no postnatal *S. aureus* challenge (n = 10); the antibiotic + Probio-M9 group, receiving both antibiotics and Probio-M9 as treatment after postnatal *S. aureus* challenge (n = 8); the antibiotic group, receiving antibiotics alone as treatment after postnatal *S. aureus* challenge (n = 7); the Probio-M9 prophylaxis group, receiving Probio-M9 as prophylaxis before postnatal *S. aureus* challenge (n = 8); and the Probio-M9 treatment group, receiving Probio-M9 as treatment after postnatal *S. aureus* challenge (n = 9). Female rats in each group were individually housed after mating. After delivery, all groups, except for the blank control group, underwent *S. aureus* infection via intra-mammary injection, with each nipple receiving an injection of *S. aureus* at a concentration of 3×10^4^ CFU/mL). In the Probio-M9 prophylaxis group, Probio-M9 gavages were administered at a dosage of 4 × 10^9^ CFU per day for 21 days. For the remaining groups, saline gavages were given from -21 days to -1 day prior to mammary gland injection. The antibiotic treatment groups received cephalexin gavages at a dosage of 150 mg per rat per day after *S. aureus* infection on days 1-3. The Probio-M9 treatment groups received Probio-M9 gavages at a dosage of 4 × 10^9^ CFU per day after *S. aureus* infection on days 1-3. In the group receiving both antibiotic and Probio-M9, cephalexin was administered at a dosage of 150 mg per rat per day), given 2 h before administering Probio-M9 at a dosage of 4 × 10^9^ CFU per day on days 1-3. After three days of treatment, the rats were euthanized, and their mammary gland tissues were collected for further analysis. All procedures were conducted under strict aseptic conditions.

In Experiment II, there were a total of six groups, each consisting of 12 female rats (Fig. 5A). The first group served as the positive control and was solely challenged with *S. aureus*. The second group, known as the blank control, did not undergo any challenges. The remaining three groups were as follows: female rats that did not mate, subjected to intramammary challenge with *S. aureus* and fed probiotics; female rats that mated, subjected to intramammary challenge with *S. aureus* and fed probiotics; and female rats that mated but were not subjected to intramammary challenge with *S. aureus* and fed probiotics.

The dosages of probiotics and *S. aureus*, as well as the timing of intra-mammary *S. aureus* challenge and euthanasia, were consistent between both experiments. Mammary gland tissues were collected from all rats in Experiment I, while both mammary gland tissues and MLN were collected from all rats in Experiment II. All procedures were performed using strict aseptic techniques.

In Experiment I, histological analysis was conducted on mammary gland tissue samples collected from all groups. For the three groups of rats that were fed Probio-M9, the mammary gland tissue was homogenized, and the resulting homogenates were plated out onto de Mann, Rogosa and Sharpe agar to recover Probio-M9. The isolated colonies were subsequently subjected to confirmation of their identity as Probio-M9 using strain-specific PCR, whole-genome sequencing, and genome assembly, as described below. In Experiment II, both samples of mammary gland tissues and MLN were homogenized and processed to recover and confirm the presence of Probio-M9.

**Hematoxylin and eosin staining**

Samples of mammary gland tissue were immediately fixed in a 10% formalin solution. The tissues were then sectioned into 5 μm thickness using a Leica RM2235 system (Leica, Wetzlar, Germany). Following sectioning, the tissues underwent a dehydration process using a series of graded ethanol and xylene solutions. Subsequently, they were embedded in paraffin, stained with hematoxylin and eosin, and visualized using a microscope according to a previous study [1]

**Detection of cytokine levels in mammary tissues**

Cytokine levels (IL-1β, IL-6, IL-4, IL-2, IL-10, and IFN-γ) were quantified using enzyme-linked immunosorbent assay kits (R&D Systems, Cambridge, MA, USA), following the manufacturer’s protocols. Briefly, 50 μL of test diluent RD1-63, standard, control, or sample were added to each well and stirred for 1 min, followed by incubation at room temperature for 2 h. Each well was then washed five times with 400 μL of washing solution. Subsequently, 100 μL of conjugate was added to each well and incubated at room temperature for 2 h. After that, 100 μL of substrate solution was added to each well and incubated at room temperature for 30 min, protected from light. Following the incubation, 100 μL of blocking solution was added to each well and thoroughly mixed. The optical density was measured in 30 min at 450 nm and 570 nm using a spectrophotometer Qubit (Thermo Fisher Scientific Inc., Waltham, Massachusetts, USA).

**Recovery of Probio-M9 and genomic DNA extraction**

The samples were homogenized, and the resulting tissue homogenates were inoculated onto de Mann, Rogosa and Sharpe agar. The agar plates were then incubated at 37 °C for 24 h to facilitate the growth of lactic acid bacteria. Owing to the low probability of isolating Probio-M9, we selected a substantial number of individual bacterial colonies that exhibited similar morphology to Probio-M9 for subsequent testing. The selected colonies were purified by streaking them onto fresh de Mann, Rogosa and Sharpe agar plates, followed by culturing them in a liquid medium at 37 °C for 24 h. Afterward, bacterial cells were harvested by centrifugation at 3000 × *g* for 8 min. Genomic DNA was extracted from the harvested bacteria using the sodium dodecyl benzene sulfonate method [2]. The extracted DNA was visualized by agarose gel electrophoresis and quantified by using a Qubit fluorometer (Life Technologies Corporation, Carlsbad, CA, USA).

**Confirmation of strain identity**

**PCR detection of Probio-M9**

The extracted DNA from each isolate was subjected to strain-specific PCR using a pair of Probio-M9-specific primers (M9F: 5’-GTAATGTAAATGGGGTTCCTGTG-3, M9R: 5’-TGGTTTCCCTATAAT CGTTGTCC-3) to confirm the identity of Probio-M9. The PCR amplification was conducted using an Applied Biosystems PCR system (Thermo Fisher Scientific Inc., Waltham, MA, USA) with an annealing temperature of 58 °C for 30 cycles. The PCR reaction mixture consisted of a 50 μL reaction system, including 25 µL of Roche Diagnostics KAPA HiFiTM HotStart Ready Mix (Roche Diagnostics Corporation, Indianapolis, IN, USA), 1.2 µL each of the forward and reverse primers, 1.5 µL of template, and supplemented with ddH2O to a final volume of 50 µL. DNA samples that yielded a positive result in the Probio-M9-specific PCR were subsequently subjected to whole-genome sequencing to further confirm their strain identity and determine the level of similarity with the originally administered Probio-M9 bacteria.

**Sequence analysis**

The same genomic DNA was used for sequencing. Illumina paired-end libraries (151 bp length, coverage 500×) were generated using the Illumina Hiseq Xten platform. The paired-end reads were subjected to de novo assembly using SOAPdenovo v2.04 software [3]. Any gaps in the assembly were filled using the Gapcloser software (available at http://sourceforge.net/proiects/soapdenovo2/files/GapCloser/). To assign bacterial taxonomy, the blastall [4] command (blastn subroutine, -e 1e-5, bite score > 2000) was used to compare the genome assembly results against the GTDB database [5]. Pair-wise average nucleic acid identity [6] and total nucleic acid identity [7] values were calculated using custom Perl scripts.

**Identification of SNPs**

The contigs of each isolate were aligned to the Probio-M9 genome to identify SNPs using MUMmer 3.0 [8]. Corresponding SNPs were selected according to the following criteria: (a) quality scores > 20, indicating an average base calling error rate of < 0.01; (b) coverage by > 10 paired-end reads; and (c) exclusion of SNPs located in repetitive regions.

**Functional annotation and pan-core genome construction**

All isolates and the original Probio-M9 were functionally annotated with Prokka software [9] using default parameters. In addition, Roary software [10] was used to analyze the pan-core genomes.

**Extraction and sequencing of** **metagenomic DNA**

Fecal samples were collected at two time points: day -26 and day 4. DNA extraction from the samples was performed using the QIAamp Fast DNA Stool Mini Kit (Qiagen, Hilden, Germany). Subsequently, sequencing libraries were generated using the NEBNext® Ultra™ DNA Library Prep Kit for Illumina (New England Biolabs, Ipswich, MA, USA) following the manufacturer’s instructions. The libraries were then sequenced on the Illumina Hiseq X-ten platform to generate 150 bp paired-end reads. In total, 744.774 Gb of raw data were generated, with an average of 6.77 Gb of data per sample.

**Reads assembly, contig binning, genome dereplication**

In this study, metagenomic bins were assembled using methods described in a previous study [11]. The reads were initially assembled into contigs using MEGAHIT software [12]. Contigs with a length greater than 2,000 bp were selected for binning using MetaBAT2 [13] with default parameters to obtain metagenome-assembled genomes (MAGs). To assess the quality of the MAGs, the reads were mapped back to their respective contigs using BWA-MEM [14]. The read depth was calculated using Samtools [15] and the jgi_summarize_bam_contig_depths function in MetaBAT2. The completeness and contamination of the MAGs were evaluated using the CheckM software [16]. The MAGs meeting the criteria of high quality (completeness ≥ 80%, contamination ≤ 5%), medium quality (completeness ≥ 70%, contamination ≤ 10%), and partial quality (completeness ≥ 50%, contamination ≤ 5%) were classified accordingly. The high-quality genomes were further clustered, and representative genomes from each replicate set were selected using dRep software [17] with the parameter settings of -pa 0.95 and -sa 0.95. Finally, a total of 307 SGBs were extracted from the analysis.

**Taxonomic annotation and abundance of SGBs**

The annotation of SGBs was accomplished using the National Center for Biotechnology Information (NCBI) non-redundant protein sequence database. The predicted genes from the SGBs were searched against the UniProt Knowledgebase (UniProtKB, released 2020.11) using the blastp function of DIAMON [18] with default settings. To estimate the abundance of each SGB, the normalized method, CoverM (available at https://github.com/wwood/CoverM), was executed with the following parameters: “-min-read-percent-identity 0.95-min-covered-fraction 0.4”.

The Kyoto Encyclopedia of Genes and Genomes Orthology Database and Carbohydrate-Active EnZyme Database were used for annotating the key metabolic genes and carbohydrate-active enzymes, respectively. Correlation analysis was used to explore the relationship between differential SGBs and differential genes using the Pearson correlation coefficient. Spearman analysis was used to explore the correlation relationship of the collinear network graph. Additionally, partial Mantel tests were conducted to evaluate correlations among cytokines, differential SGBs, and differential metabolic genes. The data were visualized in the R environment with the linkET package (version 0.0.3.7).

**Statistical analysis**

Statistically significant differences between groups (*P* < 0.05) were determined using Wilcoxon tests for comparisons between two samples or Kruskal-Wallis tests for comparisons between three or more samples. These tests were performed using R software (version 3.6.3).

For pan-genome analysis, the pheatmap package in R software was used. Principal coordinate analysis (Bray-Curtis distance) and the Adonis test (with 999 permutations) were performed and visualized using the R packages, vegan (https://github.com/vegandevs/vegan) and ggpubr (https://rpkgs.datanovia.com/ ggpubr/). All graphical presentations were generated using R software and Adobe Illustrator.


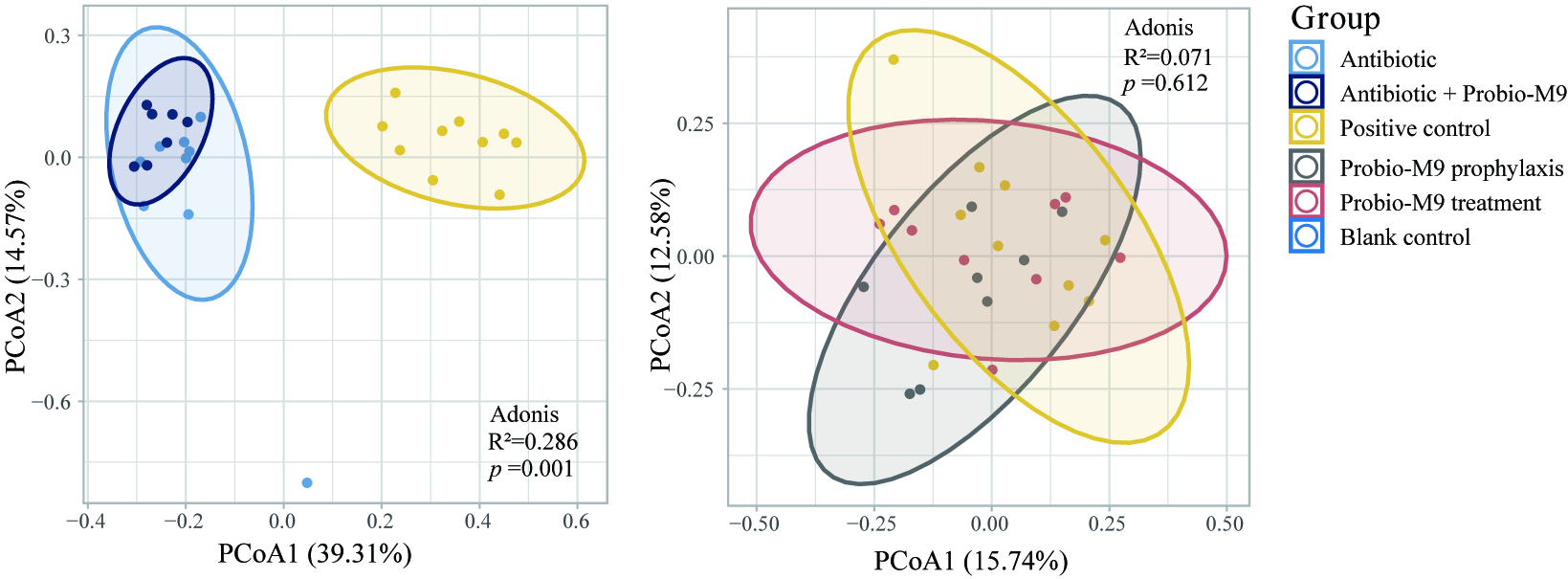


Figure S1. **Analysis of beta diversity of fecal metagenome in rats.** Principal coordinates analysis (PCoA; Bray-Curtis distance; at 4 days after intramammary bacterial challenge) score plots of positive control group, antibiotic group, and antibiotic + Probio-M9 group at 4d (left panel); positive control group, Probio-M9 treatment group, and Probio-M9 prophylaxis group. The *p* and R^2^ values of the Adonis tests are shown.


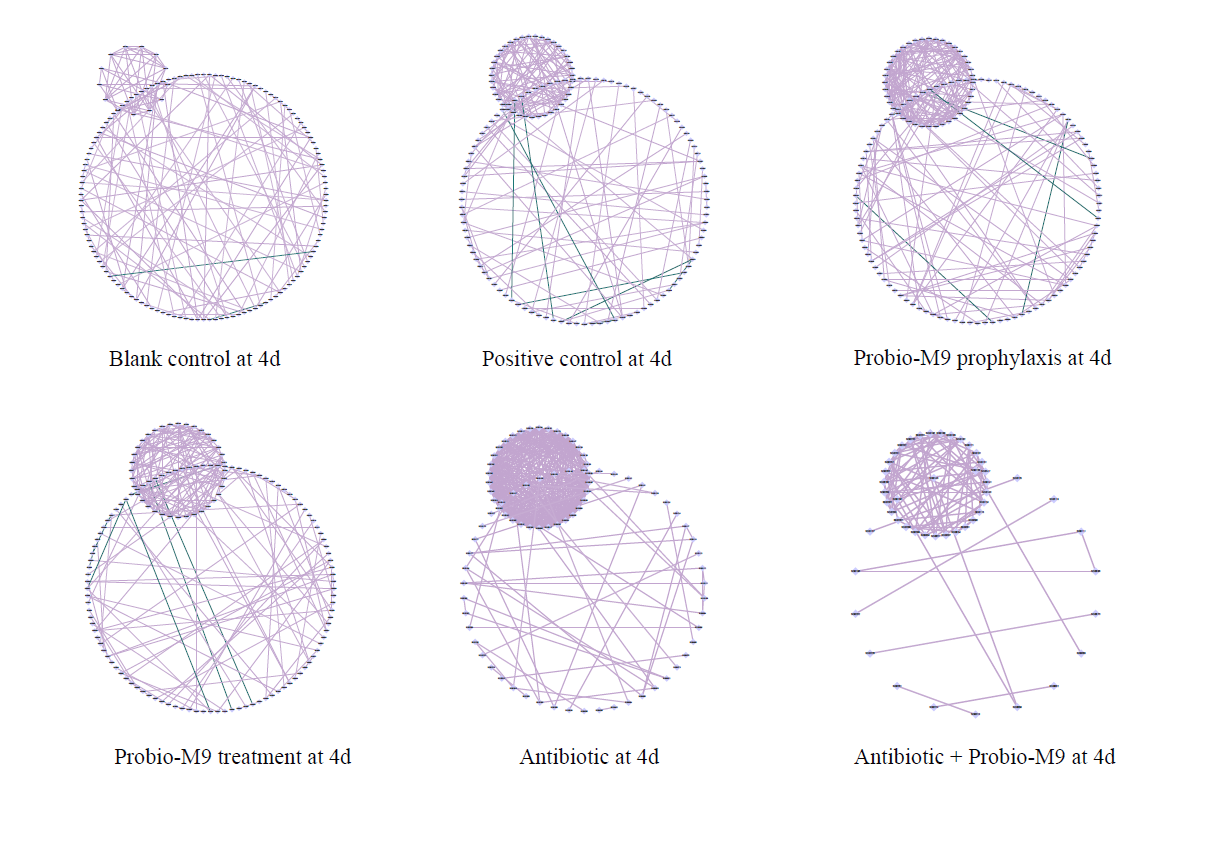


Figure S2 **Co-occurrence networks of each group at day 4 of rat fecal metagenome.** Six co-occurrence networks of each group at day 4 of the SGBs are separately illustrated. The edges between nodes represent correlations between the nodes they connect and the color purple of lines in the network refer to positively correlations and the color of green refer to negative correlations (|r| >0.8, *p* < 0.001).


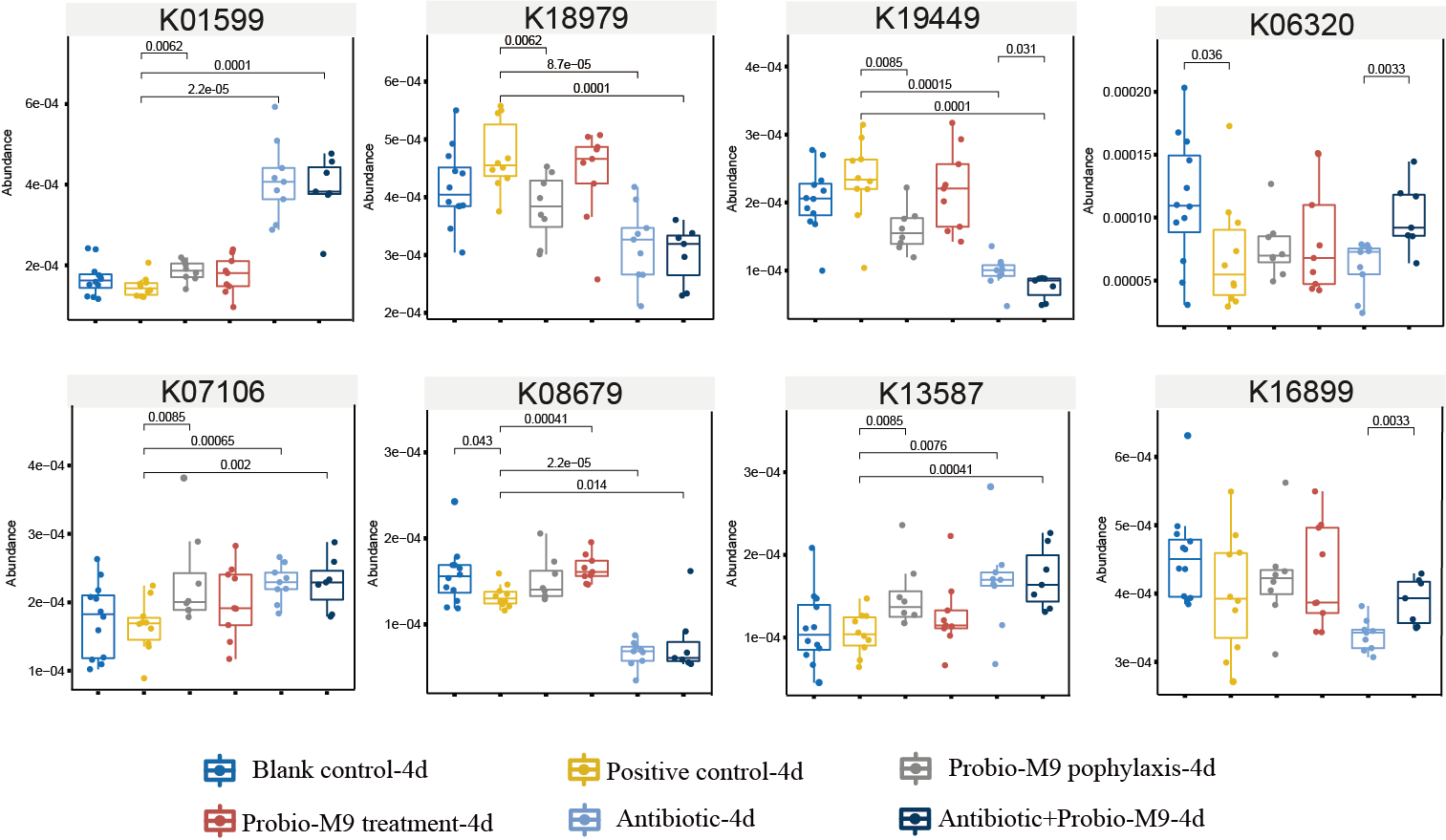


Figure S3. **Analysis of metagenomic functional gene differences of fecal metagenome in rat.** Functional gene difference of intestinal flora in different groups of rats. The analysis was conducted based on data from rats 4 days after the intramammary *Staphylococcus aureus* challenge. Boxplots showing significant differential Kyoto Encyclopedia of Genes (KEGG) between groups. All the shown KOs were not significantly different between groups at baseline. The indicated *p*-values represent statistically significant differences (cut-off level of *p* < 0.05, Wilcoxon test).


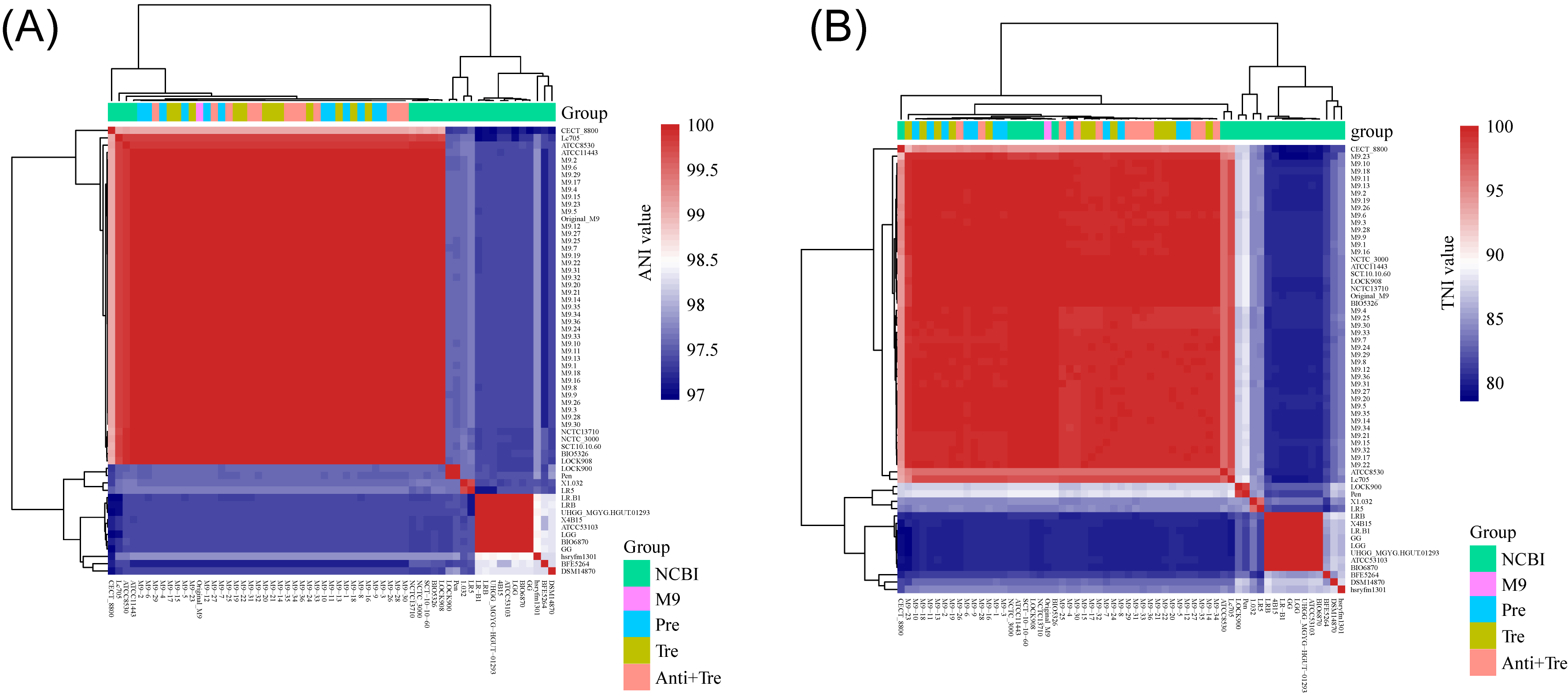


Figure. S4. **Analysis of nucleic acid diversity of Probio-M9 isolates in rats in experiment I.** Differences between Probio-M9 strain in experiment I and NCBI strain about average nucleic acid identity (A) and total nucleic acid identity (B).


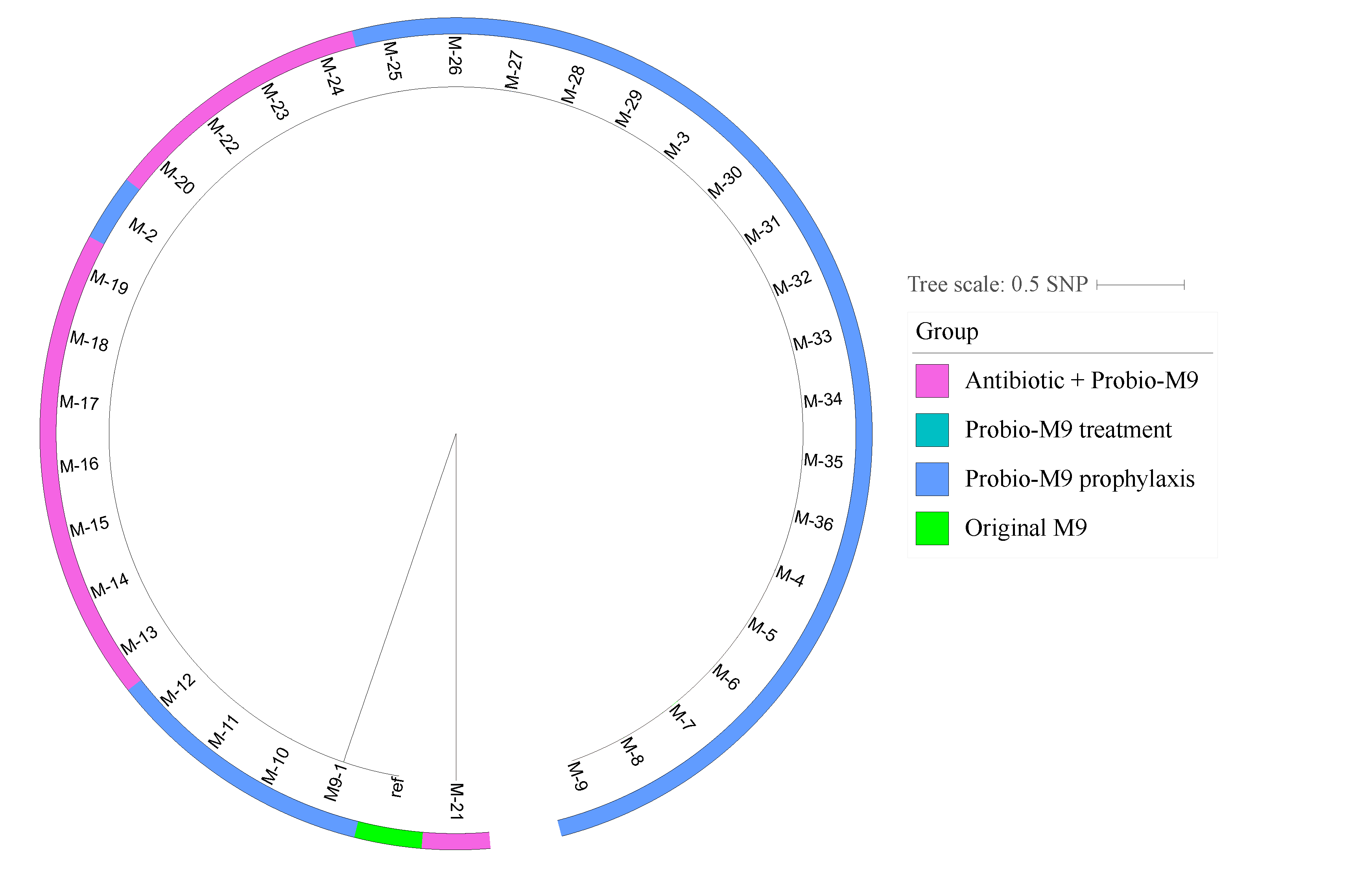


Figure S5 **Phylogenetic analysis of Probio-M9 isolates in rats in experiment I.** In Experiment I, a total of 36 Probio-M9 homologous isolates were recovered from the mammary tissue samples collected from different rat groups after euthanasia. The phylogenetic tree was constructed based on single nucleic acid polymorphism analysis, with the original Probio-M9 genome serving as the reference sequence.

Figure S6. **Analysis of nucleic acid diversity of Probio-M9 isolates in rats in experiment II.** Differences between Probio-M9 strain in experiment II and NCBI strain about average nucleic acid identity (A) and total nucleic acid identity (B).


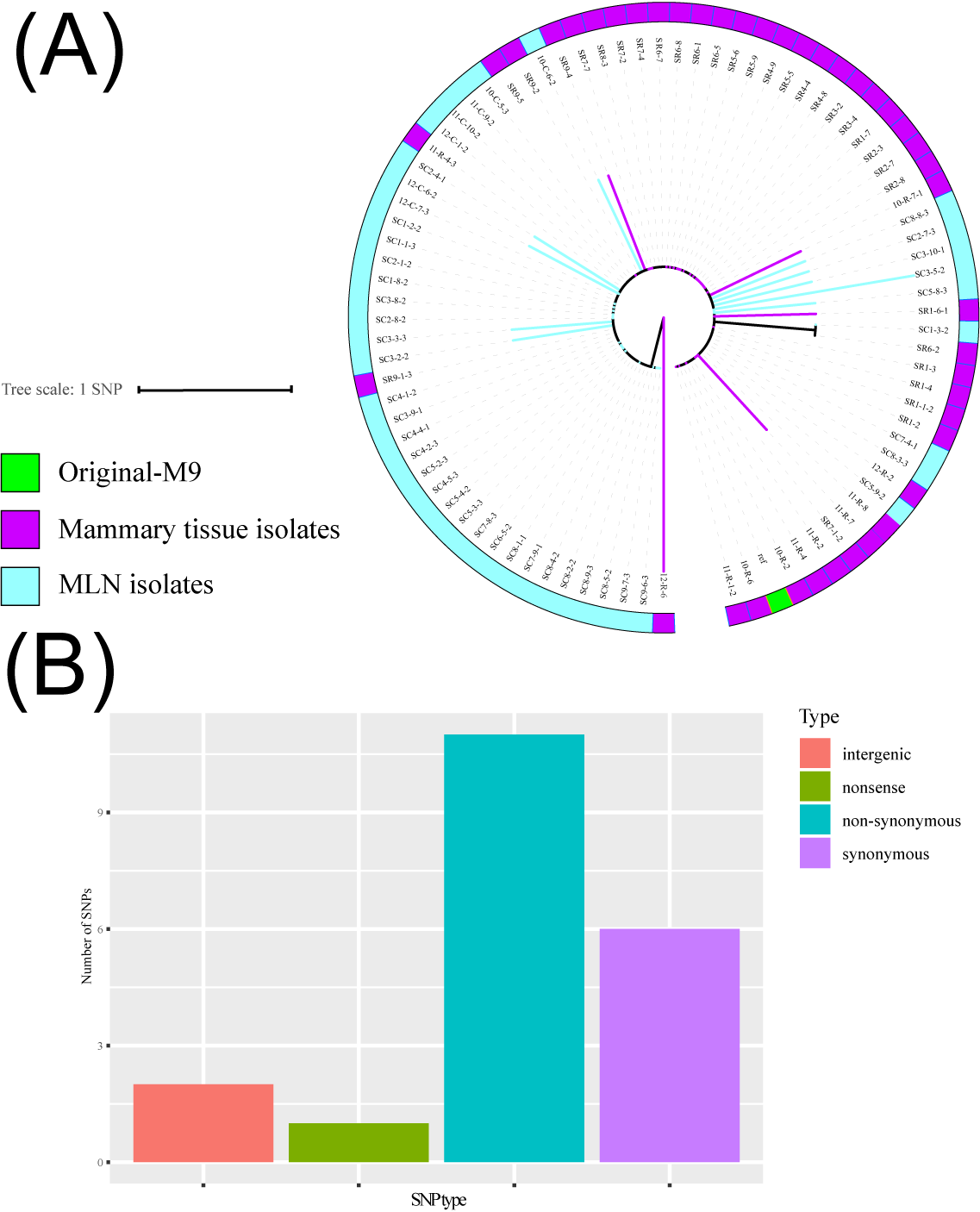


Figure S7. **Phylogenetic analysis and single nucleic acid polymorphisms (SNPs) functional annotation of rat Probio-M9 isolates based on SNPs analysis in experiment II.** (A) Probio-M9 homologous isolates were exclusively identified from the mammary tissue and mesenteric lymph node samples collected from the Probio-M9 challenged mating groups. Specifically, 42 isolates were recovered from the MLN, while 43 isolates were obtained from the rat mammary tissue samples. The phylogenetic tree was constructed using SNPs analysis, with the original Probio-M9 genome serving as the reference sequence. All Probio-M9 homologous isolates were genetically identical to the original Probio-M9 strain, with fewer than three SNPs observed across all isolates. (B) The bar chart shows the number and types of SNPs observed among the isolates.

**Reference**

1. Zhang, Yong, Qiangchuan Hou, Chen Ma, Jie Zhao, Haiyan Xu, Weicheng Li, Yanjie Wang, Huimin Ma, Heping Zhang, Zhihong Sun. 2019. “Lactobacillus casei protects dextran sodium sulfate-or rapamycin-induced colonic inflammation in the mouse.” *European journal of nutrition* 1-9.

2. Wilson, T, J Carson. 2001. “Rapid, high-throughput extraction of bacterial genomic DNA from selective-enrichment culture media.” *Letters in Applied Microbiology* 32: 326-330.

3. Luo, Ruibang, Binghang Liu, Yinlong Xie, Zhenyu Li, Weihua Huang, Jianying Yuan, Guangzhu He, Yanxiang Chen, Qi Pan, Yunjie Liu. 2012. “SOAPdenovo2: an empirically improved memory-efficient short-read de novo assembler.” *Gigascience* 1: 2047-2217X-2041-2018.

4. Ye, Jian, Scott McGinnis, Thomas L Madden. 2006. “BLAST: improvements for better sequence analysis.” *Nucleic acids research* 34: W6-W9.

5. Parks, Donovan H, Maria Chuvochina, David W Waite, Christian Rinke, Adam Skarshewski, Pierre-Alain Chaumeil, Philip Hugenholtz. 2018. “A standardized bacterial taxonomy based on genome phylogeny substantially revises the tree of life.” *Nature biotechnology* 36: 996-1004.

6. Goris, Johan, Konstantinos T Konstantinidis, Joel A Klappenbach, Tom Coenye, Peter Vandamme, James M Tiedje. 2007. “DNA–DNA hybridization values and their relationship to whole-genome sequence similarities.” *International journal of systematic and evolutionary microbiology* 57: 81-91.

7. Chen, Jiapeng, Xianwei Yang, Jianwei Chen, Zhong Cen, Chenyi Guo, Tao Jin, Yujun Cui. 2015. “SISP: a fast species identification system for prokaryotes based on total nucleotide identity of whole genome sequences.” *Infectious Diseases and Translational Medicine* 1: 30-55.

8. Kurtz, Stefan, Adam Phillippy, Arthur L Delcher, Michael Smoot, Martin Shumway, Corina Antonescu, Steven L Salzberg. 2004. “Versatile and open software for comparing large genomes.” *Genome biology* 5: R12.

9. Seemann, Torsten. 2014. “Prokka: rapid prokaryotic genome annotation.” *Bioinformatics* 30: 2068-2069.

10. Page, Andrew J, Carla A Cummins, Martin Hunt, Vanessa K Wong, Sandra Reuter, Matthew TG Holden, Maria Fookes, Daniel Falush, Jacqueline A Keane, Julian Parkhill. 2015. “Roary: rapid large-scale prokaryote pan genome analysis.” *Bioinformatics* 31: 3691-3693.

11. Sun, Baoqing, Teng Ma, Yalin Li, Ni Yang, Bohai Li, Xinfu Zhou, Shuai Guo, Shukun Zhang, Lai-Yu Kwok, Zhihong Sun. 2022. “Bifidobacterium lactis Probio-M8 Adjuvant Treatment Confers Added Benefits to Patients with Coronary Artery Disease via Target Modulation of the Gut-Heart/-Brain Axes.” *Msystems* 7: e00100-00122.

12. Li, Dinghua, Chi-Man Liu, Ruibang Luo, Kunihiko Sadakane, Tak-Wah Lam. 2015. “MEGAHIT: an ultra-fast single-node solution for large and complex metagenomics assembly via succinct de Bruijn graph.” *Bioinformatics* 31: 1674-1676.

13. Kang, Dongwan D, Feng Li, Edward Kirton, Ashleigh Thomas, Rob Egan, Hong An, Zhong Wang. 2019. “MetaBAT 2: an adaptive binning algorithm for robust and efficient genome reconstruction from metagenome assemblies.” *PeerJ* 7: e7359.

14. Li, Heng. 2013. “Aligning sequence reads, clone sequences and assembly contigs with BWA-MEM.” *arXiv preprint arXiv:1303.3997*,

15. Shmulevich, Ilya, Edward R Dougherty, Seungchan Kim, Wei Zhang. 2002. “Probabilistic Boolean networks: a rule-based uncertainty model for gene regulatory networks.” *Bioinformatics* 18: 261-274.

16. Parks, Donovan H, Michael Imelfort, Connor T Skennerton, Philip Hugenholtz, Gene W Tyson. 2015. “CheckM: assessing the quality of microbial genomes recovered from isolates, single cells, and metagenomes.” *Genome research* 25: 1043-1055.

17. Olm, Matthew R, Christopher T Brown, Brandon Brooks, Jillian F Banfield. 2017. “dRep: a tool for fast and accurate genomic comparisons that enables improved genome recovery from metagenomes through de-replication.” *The ISME journal* 11: 2864-2868.

18. Buchfink, Benjamin, Klaus Reuter, Hajk-Georg Drost. 2021. “Sensitive protein alignments at tree-of-life scale using DIAMOND.” *Nature methods* 18: 366-368.
